# Antimicrobial Properties and Assessment of the Content of Bioactive Compounds *Lavandula angustifolia* Mill. Cultivated in Southern Poland

**DOI:** 10.3390/molecules28176416

**Published:** 2023-09-03

**Authors:** Izabela Betlej, Bogusław Andres, Tomasz Cebulak, Ireneusz Kapusta, Maciej Balawejder, Sławomir Jaworski, Agata Lange, Marta Kutwin, Elżbieta Pisulewska, Agnieszka Kidacka, Barbara Krochmal-Marczak, Piotr Borysiuk

**Affiliations:** 1 Institute of Wood Sciences and Furniture, Warsaw University of Life Sciences—SGGW, 159 Nowoursynowska St., 02-776 Warsaw, Poland; boguslaw_andres@sggw.edu.pl; 2 Department of Food Technology and Human Nutrition, Institute of Food Technology and Nutrition, College of Natural Sciences, University of Rzeszów, 4 Zelwerowicza St., 35-601 Rzeszów, Poland; tcebulak@ur.edu.pl (T.C.); ikapusta@ur.edu.pl (I.K.); 3 Department of Chemistry and Food Toxicology, University of Rzeszow, 1a Ćwiklińskiej St., 35-601 Rzeszow, Poland; mbalawejder@ur.edu.pl; 4 Department of Nanobiotechnology, Institute of Biology, Warsaw University of Life Sciences, 8 Ciszewskiego St., 02-786 Warsaw, Poland; slawomir_jaworski@sggw.edu.pl (S.J.); agata_lange@sggw.edu.pl (A.L.); marta_prasek@sggw.edu.pl (M.K.); 5 Department of Plant Production and Food Safety, Carpathian State College in Krosno, 38-400 Krosno, Poland; elzbieta.pisulewska@gmail.com (E.P.); barbara.marczak@pans.krosno.pl (B.K.-M.); 6 Breeding Department, Małopolska Plant Breeding Company sp. z o. o., 4 Zbożowa St., 30-002 Kraków, Poland; akidacka@mhr.com.pl

**Keywords:** *Lavandula angustifolia*, antibacterial properties, fungicides, polyphenols, antioxidant properties

## Abstract

Lavender is a valued plant due to its cosmetic, perfumery, culinary, and health benefits. A wide range of applications is related to the composition of bioactive compounds, the quantity and quality of which is determined by various internal and external factors, i.e., variety, morphological part of the plant, and climatic and soil conditions during vegetation. In the presented work, the characterization of antimicrobial properties as well as the qualitative and quantitative assessment of bioactive compounds in the form of polyphenols in ethanol extracts from leaves and flowers of *Lavandula angustifolia* Mill. intended for border hedges, cultivated in the region of southern Poland, were determined. The composition of the fraction of volatile substances and antioxidant properties were also assessed. The conducted research shows that extracts from leaves and flowers significantly affected the viability of bacterial cells and the development of mold fungi. A clear decrease in the viability of bacteria and *C. albicans* cells was shown in the concentration of 0.32% of extracts. Leaf extracts were characterized by a much higher content of polyphenols and antioxidant properties than flower extracts. The composition of volatiles measured by GC-MS was significantly different between the extracts. Linalyl acetate and ocimene isomers mix dominated in flower extracts, whereas coumarin, γ-cadinene, and 7-methoxycoumarin were identified as dominant in leaf extracts.

## 1. Introduction

Lavender (*Lavandula angustifolia* Mill.) is one of the most valued plants used in cosmetology and aromatherapy. It has a deodorizing, relaxing, antiseptic, and anti-inflammatory effect. In the Mediterranean countries, it is used in the design of creative cuisine as an additive that improves the taste of meat, ice cream, and drinks and also as a food preservative. Lavender is also a highly valued plant in landscaping, as it is suitable for borders, hedges, and decorations. Scientific research also indicates the ability of the plant to phytoremediate the soil from pollutants, especially heavy metals [1].

The most valuable part of the plant are the flowers, which contain up to 4.5% of the essential oil. The main components of the oil are esterified and free linalool, camphor, and cineol [2]. Lavender leaves typically contain more camphor and cineole than the flowers, making the leaf oil more pungent. In addition to the oil, compounds such as tannins, phenolic acids, flavones, anthocyanidins, saponins, polyphenols, and minerals have been identified in the chemical composition of the plant. According to Tăbăraşu et al. [3], essential oil can contain up to 300 different compounds, the qualitative and quantitative composition of which depends on the species, morphological part of the plant, and environmental factors affecting yield. Barbieri and Borsotto [4], in their research, indicate that younger plants synthesize more essential oil than older plant seedlings. In older plants, however, the oil is characterized by a richer chemical composition. Najar et al. [5] showed correlations between the content of linalool, 3-octanone, and α-terpineol and the age of the plant. Similar relationships were shown by Détár et al. [6] in six varieties of Lavandula angustifolia during the two-year harvest. The authors of the study considered the main factor of this change to be the abundant amount of precipitation in one of the harvest periods of this species.

It should also be mentioned that the quality of the essential oil depends on the method of obtaining it [7]. The supercritical CO_2_ extraction method yields more oxygenated compounds, whereas the simpler and easier steam distillation method in oils yields more terpene hydrocarbons [8]. 

According to Aprotosoaie et al. [9], the genus *Lavandula* includes 39 species and about 400 varieties, however, the most popular and at the same time the most valued species is *Lavandula angustifolia*, due to its balanced chemical profile affecting the desired sensory characteristics [10]. The literature on the subject states that the main chemical components of *L. angustifolia* oil, apart from linalyl acetate and linalool, are β-linalyl and myrcene, and in lavender grown in Poland—geraniol and bornyl acetate [11,12]. Caprari et al. [13] indicated that the cultivation and industrial use of L. angustifolia is the largest among the known and available lavender species cultivated in Italian regions, and the most valued. The substances contained in *L. angustifolia* give the plant and its extracts antibacterial and fungicidal properties [14,15]. Adaszyńska-Skwirzyńska et al. [16] proved the synergistic effect of combinations of fuconazole and oils obtained from the flowers and leaves of *L. angustifolia* against the *Candida albicans* fungus. The fungicidal activity of lavender extracts was also found against fungi belonging to the Basidiomycetes [17], which may be important information when designing biocides to protect wood and other cellulosic materials against decomposition caused by this group of fungi. Lavender vapors are strong inhibitors of sporulation of *Trichophyton mentagrophytes* and *Trichophyton rubrum* [18]. It is therefore possible to use lavender vapors as a substance for air disinfection in rooms. 

The strong bactericidal effect of essential oils obtained from lavender was found by Flores et al. [19]. In the presented studies, the authors of the study showed that the oil with the composition of 1,8-cineole (46.5%) and camphor (12.3%) inhibited the growth of *Escherichia coli* and *Mycobacterium smegmatis*. Studies by other authors have shown that *L. angustifolia* oil in the amount of less than 1% has antibacterial activity against MRSA [20] and VRE [21]. The research conducted by Cavanagh and Wilkinson [22] showed that the essential oil obtained from lavender can be used prophylactically in the case of local bacterial infections. The antibacterial effect of lavender oil was also confirmed against *Salmonella* sp., *Enterobacter* sp., and *Klebsiella* sp. [23,24]. 

The quality of plant raw materials, including their antimicrobial and antioxidant properties, largely depends on the level of bioactive components, which in turn depend on climatic and soil conditions. Pokajewicz et al. [25] indicated that depending on climatic and soil conditions, different yields of essential oils can be obtained. *Lavandula × intermedia* from cultivation in Spain was characterized by an oil yield of 0.2–1.3% [26] and from 4.5 to 9.7% of essential oil from those grown in France [9].

The aim of the presented work was to determine the antibacterial and fungicidal properties of ethanol extracts from the flowers and leaves of *L. angustifolia* grown in southern Poland (Wyżyna Miechowska). At the same time, the chemical composition of volatile substances, polyphenol profile, and antioxidant properties were analyzed in order to confirm the relationship between the composition of the extracts and the obtained antimicrobial properties. Lavender is not a plant popular in cultivation in Poland on a large scale for use as a raw material by the cosmetic or pharmaceutical industry; it rather appears only in home architecture, in gardens. We established a large experimental crop in order to demonstrate that this plant obtained from Poland also has good antimicrobial and antioxidant properties, which could be successfully used by the pharmaceutical and domestic market of cosmetic products as an ingredient in the formulation of these products, giving them antimicrobial and antioxidant properties.

## 2. Results

### 2.1. Antimicrobial Activity

**Figure 1:**
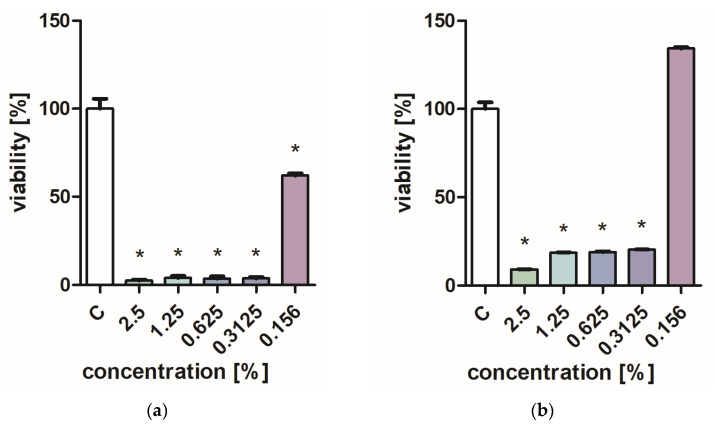

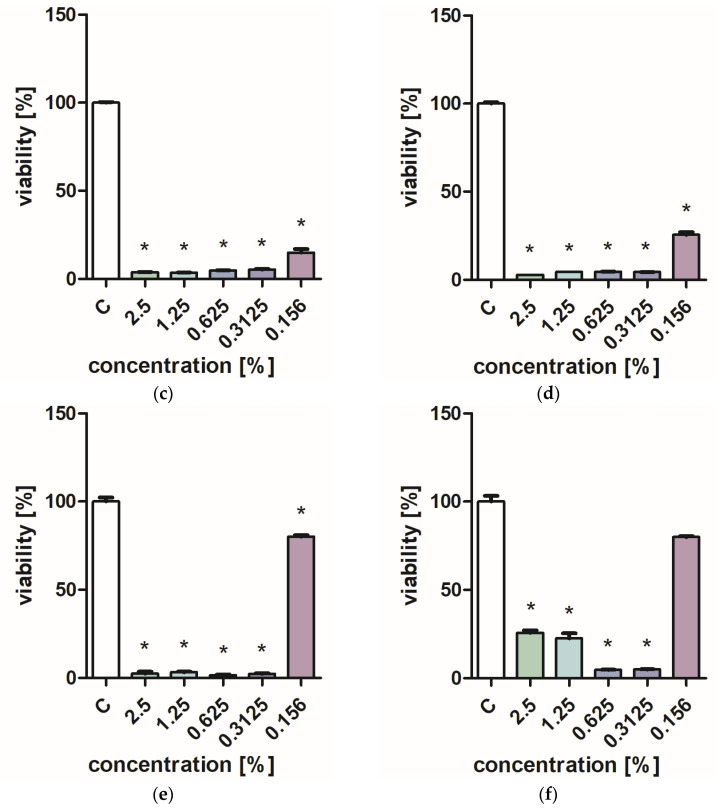
Effect of ZO1 extracts on the viability of microorganisms: (**a**,**b**) *C. albicans*; (**c**,**d**) *P. aeurginosa*; (**e**,**f**) *S. aureus*; XTT test (**b**,**d**,**f**); and PrestoBlue test (**a**,**c**,**e**). Results are presented as mean values ± standard deviation. Asterisks indicate statistical significance level at *p*-value ≤ 0.05.

**Figure 2:**
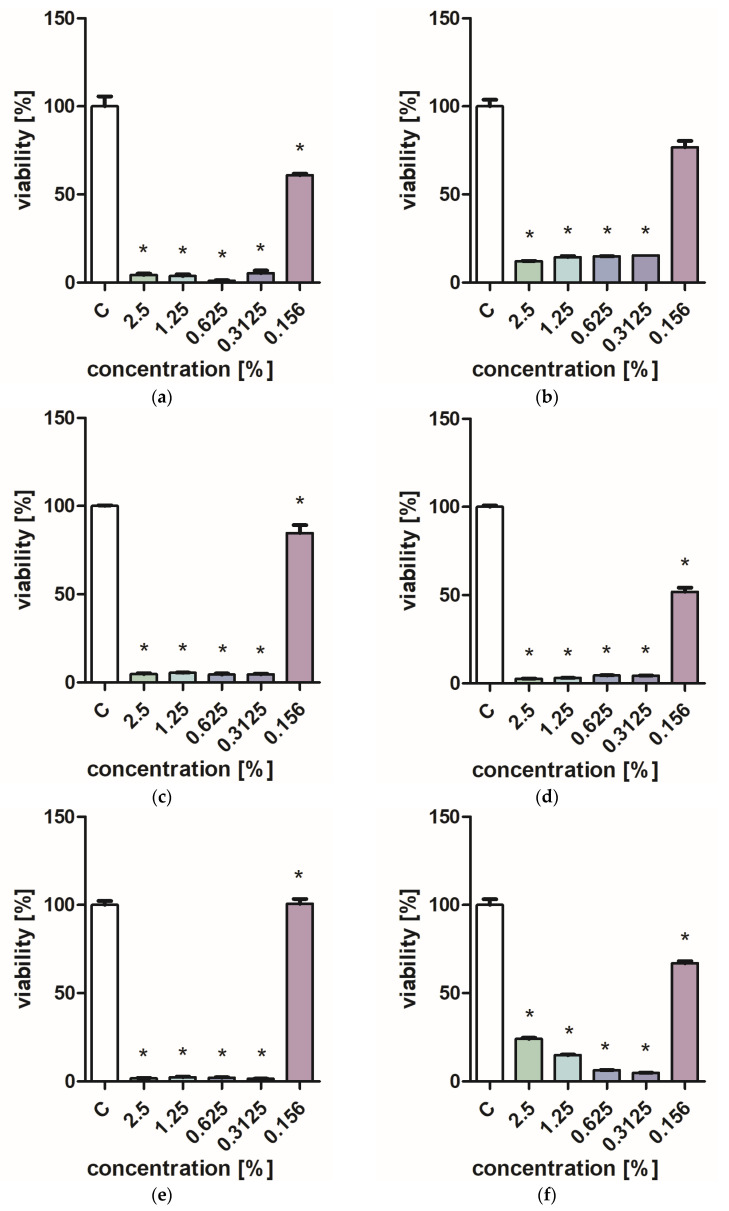
Effect of ZO2 extracts on the viability of microorganisms: (**a**,**b**) *C. albicans*; (**c**,**d**) *P. aeurginosa*; (**e**,**f**) *S. aureus*; XTT test (**b**,**d**,**f**); and PrestoBlue test (**a**,**c**,**e**). Results are presented as mean values ± standard deviation. Asterisks indicate statistical significance level at *p*-value ≤ 0.05.

Figure 1 and Figure 2 characterize the effect of lavender flower and leaf extracts on the viability of *Stapchylococcus aureus* and *Pseudomonas aeruginosa* bacteria and *Candida albicans* yeast. In each of the analyzed cases, it was found that the extracts inhibited the viability of microorganisms. Similar results were obtained in both the XTT and Presto Blue tests, indicating that extracts in very low concentrations of 0.31% are sufficient to significantly reduce the viability of microorganisms. It was also shown that lavender extracts inhibit the growth activity of mold fungi. The *Trichoderma viride* fungus is one of the organisms characterized by dynamic growth, as can be observed in control tests, in which complete overgrowth of the microbial substrate was obtained on the third day of cultivation. The growth of the tested fungus was completely inhibited when the extract content in the medium was at the level of 1 mL/100 mL (Table 1). The *Chaetomium globosum* fungus was more sensitive than *T. viride* to the biocidal effect of lavender extracts. No growth was observed on the microbial medium for any of the concentrations used (Table 2). In order to confirm or exclude the biocidal effect of 60% ethanol on the growth of fungi, control tests were performed. It was not found that the ethanol used in amounts from 0.5 to 5 mL had an inhibitory effect on the growth of the tested microorganisms.

### 2.2. Antioxidant Assay

The antioxidant activity of extracts from lavender leaves and flowers was assessed on the basis of the ability of the extracts to reduce ABTS^•+^, DPPH^•^ radicals, and TPTZ compound (FRAP). Total polyphenol content (TPC) was also measured. In all analyzed cases, a higher antioxidant activity and a higher content of polyphenols were found in lavender leaf extracts (ZO2) (Table 3). The ability of lavender leaf extracts to reduce free radicals and iron was almost twice as high as that of extracts obtained from flowers. At the same time, a very high level of influence (99.9%) of the type of extract (ZO1 and ZO2) on their antioxidant activity was indicated (Table 4). The error in this respect was only 0.1%, which proves the negligible influence of other factors, which were not taken into account in the conducted research. With regard to the content of polyphenols, a significant effect of the type of extract was also noted, amounting to 98.9%.

### 2.3. Chemical Composition

GC-MS analysis showed clear differences in the chemical composition of leaves and flowers. The predominant component of the flowers turned out to be linalyl acetate, whose percentage in the extract was estimated at 25.83% (Table 5 and Table 6). A sizable content of ocimene isomers belonging to monterpenes was also identified. Linalool, which dominates the composition of essential oils, was identified at the level of 2.23% in the obtained ethanol extracts. Small amounts of β-pinene (2.39%), limonene (7.09%), and myrcene (4.79%) have also been identified in flower extracts. Eleven substances were identified in lavender leaf extracts, and only four of the identified substances coincided with the substances identified in flower extracts. The leaf extracts were dominated by coumarin, γ-cadinene, 7-methoxycoumarin, and α-santalene (Table 7 and Table 8). The identification of p-cymene, m-cymene, limonene, and eucalyptol were within detection limits. The confirmation of correctly identified compounds is the calculated linear retention indexes (LRI). In addition to the calculated LRI, the index values given in the literature have also been added for direct comparison and to verify the accuracy of the identification.

Also, it should be stated that in the evaluation of the content of phenolic compounds (HPLC), leaf extracts are characterized by a higher content of these components. Based on the conducted research, 22 substances belonging to phenolic compounds were characterized (Table 9). Rosmarinic acid and ferulic acid glucosie III were dominant in both leaf and flower extracts. In addition, very high amounts of undefined caffeic acid derivative were identified in lavender leaf extracts. In the case of flower extracts, only two of the identified substances—chicoric acid and apigenin—were present in higher amounts than in leaf extracts. 

### 2.4. Graphical Interpretation of Results

**Figure 3:**
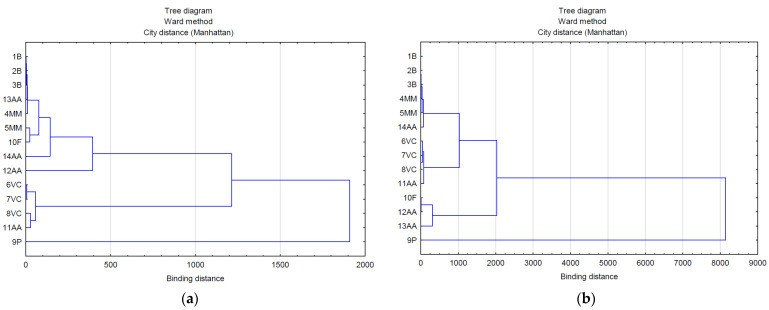
Analysis of the relationship between the marked features in extracts from: (**a**) flowers (ZO1) and (**b**) leaves (ZO2).

**Figure 4:**
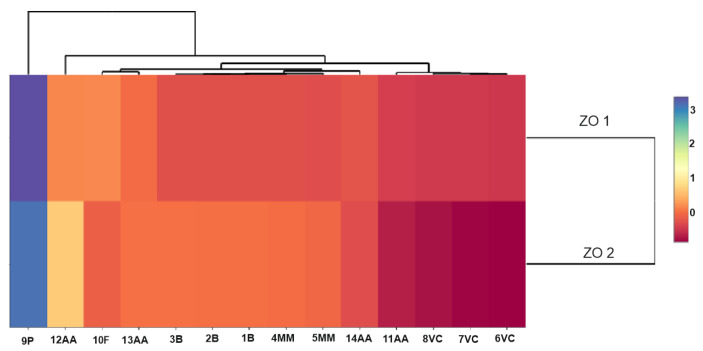
Scaled heat map of extracts from leaves (ZO2) and flowers (ZO1).

The use of multivariate analysis (clusters) made it possible to capture the hidden relationships between the analyzed variables within leaf extracts and do so separately from lavender flower extracts (Figure 3a,b). The applied data agglomeration showed connections between antimicrobial properties (antibacterial, fungicide) and the content of volatile compounds, phenolic acids, and flavonoids. The cluster analysis of compounds extracted from lavender flowers (ZO1) (Figure 3a) formed three main clusters, of which phenolic acids (9P) were in the first one and volatile compounds represented by the variables 6VC, 7VC, 8VC, and 11AA in the second. The third cluster included the remaining analyzed data (1B, 2B, 3B, 13AA, 4MM, 5MM, 10F, 14AA, 12AA). The graphical interpretation of the data resulting from the cluster analysis in the case of lavender leaf extracts (ZO2) (Figure 3b) also showed three main data clusters characterized by common variability. Within the first cluster, as in the case of ZO1, there were phenolic acids, whereas the second cluster contained antioxidant activity expressed by the ABTS test (12AA), the DPPH test (13AA), and the content of flavonoid compounds (10F). The remaining data were included in cluster 3. The use of a heat map with the scaling function allowed for a graphical comparison of the qualitative characteristics of both types of extracts (Figure 4). It is clearly visible that lavender leaf extracts (ZO2) have a higher antioxidant activity expressed by TCA (11AA), ABTS (12AA) and FRAP (14AA) tests, and a higher content of phenolic acids (9P), flavonoids (10F), and volatile compounds than lavender extracts from ZO1 lavender flowers. Lavender flower extracts, on the other hand, have a slightly higher antibacterial activity than lavender leaf extracts.

## 3. Discussion

In recent years, there have been there have been an increasing number of detailed scientific reports on the assessment of the properties of lavender raw material. This plant, due to its health care and culinary values, is more and more often cultivated in various regions of the world. In the studies of many authors, the quality of plant materials largely depends on climatic and soil conditions [27,28]. In our own research, it was found that extracts from lavender leaves and flowers obtained from cultivation in a temperate climate on very good wheat complex soils are characterized by good antibacterial and antifungal properties. The effect on the viability of microorganisms obviously depends on the dose of the extract, however, in this study, it was proved that small concentrations of the extract, amounting to about 0.32%, were sufficient to significantly reduce the viability of bacteria and fungi of the genus *Candida*. High activity inhibiting the growth of bacterial cells was also demonstrated by other researchers [29,30,31]. In turn, Adaszyńska et al. [32] found that three out of five varieties of lavender grown in Poland do not inhibit the growth of *P. aeruginosa*. Literature data indicate that the essential oil is an effective fungicide. Cavanagh and Wilkinson [33] found that *L. angustifolia’s* oil is effective against *Aspergillus niger* and *Trichophyton mentagrophytes*. Also, in this study, it was proven that other types of extracts effectively inhibit the development of mold fungi. Different sensitivity of fungi to extracts was shown. The *Trichoderma viride* fungus turned out to be more resistant to the growth-inhibiting effect of lavender leaf and flower extracts than the *Chaetomium globosum* fungus. Similarly, different sensitivity of fungi to the tested lavender oils was shown by Caprari et al. [34].

Antibacterial and antifungal properties are determined by the composition of chemical components contained in the extracts. Significantly higher amounts of phenolic compounds were identified in lavender leaf extracts than in flower extracts. At the same time, using a statistical cluster analysis diagram, it was shown that there is a relationship between the antimicrobial activities and the presence of specific phenolic compounds in the extracts. According to Tăbăraşu et al. [3], the quantity and quality of phenolic compounds in the plant material depends on both the ambient temperature, the season, the nutrients in the soil, and the anatomical part of the plant. The total phenol content in the leaf extracts was 31.64 mg GAE/g and was less than half that of the flowers. Obviously, the extraction methods also have a significant impact on the final quality of the extract. Costa et al. [35] showed that water and an ethanol–water mixture are the most suitable solvents for the extraction of phenolic compounds. In turn, Dobros et al. [36] confirmed that the type of extraction, temperature, and ratio of solvent to sample may affect the ability to extract polyphenolic compounds. The obtained lavender extracts were rich in phenolic compounds such as rosmarinic acid, ferulic acid glucosie III, or coumaric acid glucoside I. Similar results were obtained by other researchers [37]. The presented analyzes showed significant differences in the content of some phenolic compounds in individual types of extracts. The content of caftaric acid in leaf extracts was 10 times higher than in lavender flower extracts. In turn, the content of chicoric acid was almost three times higher in flower extracts. The richer the quantitative composition of phenolic compounds in leaf extracts, the higher the number of antioxidant properties observed. The antioxidant activity of flower extracts in the DPPH radical method and the FRAP method was 97.47 and 62.88 µmol Trolox/g, respectively, and was similar to the antioxidant activity of *L. angustifolia* flower extracts determined by Dobros et al. [36]. In the studies conducted by other authors, the antioxidant activities of ethanol extracts from lavender flowers also oscillated at a similar level [38].

In this study, differences in the qualitative and quantitative composition of volatile substances extracted from various morphological parts of plants were identified. Small amounts of linalool were identified in flower extracts, which, according to literature data, is the dominant component of essential oils from lavender flowers [39]. The difference identified in the linalool content between the essential oil and the HS volatile fraction of the flower extract can be attributed to the relatively lower volatility of terpene alcohols (such as linalool) when compared to hydrocarbons with an equivalent number of carbon atoms. According to literature data, this ingredient also has a biocidal effect against *C. albicans* fungi [40]. The dominant component in HS fraction of the flower extracts was linalyl acetate due to the higher vapor pressure of these compounds compare to linalool In addition, compounds belonging to sesquiterpenes and monoterpene hydrocarbons, such as pinene, myrcene, carene, and santalene, have also been identified. According to Pokajewicz et al. [25], the composition of essential oil substances, even within the same species, depends on many factors, such as the place of cultivation, weather conditions, or harvest time. However, it should be noted that the qualitative composition of ethanol extracts from flowers was similar to the composition of volatile substances identified in oils from flowers of the *L. angustifolia* cultivar [41,42]. The lavender leaf extracts were dominated by coumarin, cadinene, and methoxycoumarin. Hajhashemi et al. [43] also indicate that the dominant components in the above-ground parts of the plant are ursolic acid and coumarins.

## 4. Materials and Methods

### 4.1. Plant Material

The material for the study was one of the three ecotypes of narrow-leaved lavender (*Lavandula angustifolia* Mill.)—a dwarf form with short stems used for low border hedges (ZO) and cultivated in southern Poland. The field experiment was carried out in Poland, in Polanowice (50°19′ N 20°07′ E), a farm belonging to the Małopolska Plant Breeding. The research was carried out on chernozem (valuation class 1), with a calcareous substrate (pH 6.8). Seedlings obtained generatively (from seeds) were planted on 19 June 2019, in 9-point strips, 0.5 × 0.5 m apart. Mineral fertilization was applied in the spring, before planting. During the lavender growing season at the turn of March and April, spring pruning was performed (2020 and 2021), and faded inflorescences were removed. From three-year-old plants (7 July 2021), flowers and leaves, each weighing 100 g, were collected. Fresh flowers and leaves were vacuum packed and sent for analysis. The course of weather in the growing season in 2019-2021 was varied. The years 2019 and 2020 were similar in terms of precipitation (646.3 and 652.75 mm respectively), and in 2021, there was 200 mm more (845 mm). Their distribution was also different. In 2019, the most rain occurred in May and August, in 2020 in May and June, and in 2021 in July and August. In turn, the warmest year was 2019, and the highest average temperatures occurred in June, July, and August.

#### Preparation of Ethanol Extracts

The amount of 10 g of lavender (flowers and leaves separately) was flooded with 200 cm^3^ of 60% ethanol (PPS Polmos SA, Warsaw, Poland) and shaken for 72 h on a laboratory shaker (IKA KS 3000 icontrol, IKA-Werke GmbH & Co.KG, Staufen, Germany). After this time, the prepared extract was separated from lavender and then sterilized by syringe filters with a pore diameter of 0.22 µm. Flower extracts were marked with the symbol ZO1 and leaf extracts with the symbol ZO2.

### 4.2. Methods

#### 4.2.1. Evaluation of Fungicidal Properties against Mold Fungi on a Malt-Agar Medium

The effect of the extracts on the growth of mold fungi was carried out in Petri dishes on a maltose agar medium. Two species of fungi were used in the study: *Trichoderma viride* Pers., strain A-102 and *Chaetomium globosum* Kunze, strain A-141 (ATCC 6205), coming from the collection of pure cultures of the Department of Wood Science and Wood Preservation, Warsaw University of Life Sciences. Ethanol extracts of lavender in the amounts of 0.5, 1.0, 2.5, and 5 cm^3^ were added to a sterile Petri dish, followed by an amount of maltose agar medium, so that the total volume of the mixture was 10 cm^3^. Fungi were inoculated centrally into Petri dishes 24 h after gelation of the medium. 60% ethanol was used as a control. The size of the inoculum was 5-6 mm. Cultivation was carried out in a Thermolyne Type 42,000 model thermal incubator (ThermoFisher Scientific, Waltham, MA, USA) under temperature and relative humidity conditions of 26 ± 2 °C and 65 ± 2%, respectively. The assessment of fungicide properties on the culture medium was carried out by measuring the diameter of mycelial growth in two perpendicular directions. Measurements were made at 48 h intervals. The tests were completed on the day of complete overgrowth of the Petri dish in the control samples. The analysis of variance using the Snedecor statistics was used to verify the statistical analysis. Statistical inference was carried out for the significance level α = 0.05. In case of rejection of the null hypothesis, Tukey’s test was performed.

#### 4.2.2. Evaluation of Bactericidal and Anti-Yeast Properties

Microbial strains (*P. aeruginosa* (ATCC 27,853), *S. aureus* (ATCC 25,923), and *Candida albicans* (ATCC 10,231) were cultured in Mueller–Hinton broth medium (BioMaxima, Lublin, Poland) and incubated in a shaking incubator at 37 °C overnight. Before the experiments, the microbial cells were adjusted to a dedicated concentration by dilution in a sterile, distilled saline solution based on the McFarland scale.

Extracts ZO1 and ZO2 at a concentration of 5% were diluted in a 24-well plate in culture medium to concentrations of 2.5, 1.25, 0625, 0.3125, and 0.156%. Then, 10 mL of the suspension of the tested microorganisms (OD = 0.5 on the McFarland scale) was added to each well. After 24 h of incubation at 37 °C in a rotary incubator, cells from each well were harvested and centrifuged (2000 rpm, 10 min). The supernatant was removed, and the microorganisms were suspended in 1 mL PBS and pipetted into a 96-well plate. Two viability tests were carried out on the suspensions of microorganisms prepared in this way: XTT (Cell Proliferation Kit II, Merck, Darmstadt, Germany) and Presto Blue (PrestoBlue™ Cell Viability Reagent, Invitrogen, Waltham, MA, USA), according to the manufacturer’s recommendation.

#### 4.2.3. Antioxidants Properties

The total content of phenolic compounds was assessed by the method described by Gao et al. [44]. Distilled water, Folin-Ciocalteau reagent, and 20% sodium carbonate solution were added to the extracts. After 1 h, the absorbance at 765 nm was measured with a UV-VIS spectrometer (Type UV2900, Hitachi Ltd., Chiyoda, Tokyo, Japan). The results were expressed in mg GAE/g.

The scavenging activity of extracts on ABTS^•+^ radicals was determined according to the method of Re et al. [45]. The solution of ABTS^•+^ (diluted with distillated water to an absorbance of 0.7) was added to the extracts. After 6 min, the absorbance at 734 nm was measured with a spectrophotometer. Results are expressed as μmol Trolox equivalent (TE)/g.

The scavenging activity of flower extracts on DPPH˙ radicals was determined according to the method of Blois [46]. Briefly, 2.0 mL of a methanolic DPPH solution (0.1 mM) was mixed with plant extracts and left for 10 min. After this time, the absorbance was measured at 517 nm. Results are expressed as μmol Trolox equivalent (TE)/g.

The ferric ion reduction by FRAP assay was determined by the method described by Benzie and Strain [47]. Three mL of FRAP solution were added to 0.5 mL of the sample. The absorbance was recorded at a wavelength of 593 nm after 10 min of reaction. Results are expressed as μmol Trolox equivalent (TE)/g.

#### 4.2.4. GC-MS Analysis

The prepared sample was analyzed by SPME solid-phase microextraction using 100 µm polydimethylsiloxane (PDMS) fiber (Supelco Ltd., Bellefonte, PA, USA). Prior to the analyses, the fiber was conditioned according to the manufacturer’s instructions at 250 °C for 30 min in a gas chromatograph dispenser. The tested material was placed in a 100 mL conical flask secured with aluminum foil. The fiber exposure was carried out using the over-surface method for 30 min at 20 °C. Then, the fiber was transferred to the gas chromatograph injector (temp. 250 °C), where the analytes were thermally desorbed for 5 min. Using a gas chromatograph (GC-MS, Varian 450GC compressed with 240 MS), the composition of compounds desorbed from the SPME fiber was examined. Helium was the carrier gas used, the flow rate of which was 1 mL/min. The dispenser temperature was 250 °C. Separation of the analytes was carried out using a 30 m × 0.25 mm capillary column with a moderately polar HP-5 (polysiloxanmethylphenyl) stationary phase and a layer thickness of 0.25 µm. The column oven temperature program was as follows: start—50 °C for 5 min isotherm, then set to a temperature gradient of 10 °C/min to 300 °C (5 min isotherm). Based on NIST.08 and the Willey database, compounds found in the extracts were identified. GC-MS analysis was performed in duplicate.

#### 4.2.5. Phenolic Compound Identification

Polyphenolic compounds were analyzed by reverse-phase, ultra-performance liquid chromatography (UPLC)-PDA-MS/MS Waters ACQUITY (Waters, Mil-ford, MA, USA) consisting of binary pump manager, sample manager, column manager, photodiode array detector (PDA), and tandem quadrupole mass spectrometer (TQD) with electrospray ionization (ESI). Separation was performed using a BEH C18 column (100 mm × 2.1 mm id, 1.7 µm, Waters USA) maintained at 50 °C. The following solvent system was used for the study of anthocyanins: mobile phase A (2% formic acid in water *v*/*v*) and mobile phase B (2% formic acid in 40% ACN in water *v*/*v*). For the remaining polyphenolic compounds, a lower concentration of formic acid (0.1% *w*/*w*) was used. The gradient program was set as follows: 0 min 5% B, 0 to 8 min linear to 100% B, and 8 to 9.5 min for rinsing and return to baseline conditions. The injection volume of the samples was 5 µL (partial loop with needle overflow), and the flow rate was 0.35 mL/min. The following parameters were used for the TQD: capillary voltage, 3.5 kV; con voltage, 30 V in positive and negative mode; the source was kept at 120 °C; the desolvation temperature was 350 °C; gas flow con, 100 l/h; and desolvation gas flow, 800 l/h. Argon was used as the collision gas at a flow rate of 0.3 mL/min. Detection and identification of polyphenolic compounds was based on specific PDA spectra, mass-to-charge ratios, and ion fragments obtained after collision-induced dissociation (CID). Quantification was achieved by injecting solutions of phenolic compounds with known concentrations ranging from 0.05 to 5 mg/mL as standards [28,48]. All assays were performed in triplicate and expressed as µg/g. Waters MassLynx v.4.1 software was used for data acquisition and processing.

#### 4.2.6. Statistical Analysis

Statistical analysis of the results was carried out in Statistica version 13 (TIBCO Software Inc., Palo Alto, CA, USA). Analysis of variance (ANOVA) was used to test (α = 0.05) for significant differences between factors. A comparison of the means was performed by Tukey test, with α = 0.05. In order to describe the mutual relations between the examined variables, variable reduction techniques based on cluster analysis and scaled heat maps made in the R studio program were used. For easier interpretation of the results, the following codes were assigned to individual measurable features: phenolic acids (9P) and flavonoids (10F); volatile compounds: 6VC, 7VC, and 8VC; antioxidant properties: (11AA), ABTS (12AA), DPPH (13AA), and FRAP (14AA); bacteria: 1B and 2B; yeast-like fungi: 3B; mold fungi: 4MM and 5MM.

## 5. Conclusions

Extracts obtained from the flowers and leaves of *Lavandula angustifolia* grown in southern Poland were characterized by good antibacterial and fungicidal properties. Extracts from leaves and flowers showed different antioxidant properties, with the determined anti-free radical activity being higher for extracts from lavender leaves. The content of volatile substances, phenolic acids and flavonoids, as well as their activity, differs between the extracts. Based on the conducted research, it should be concluded that leaf extracts are characterized by a higher activity of biologically active compounds. The results confirm that the areas of southern Poland allow for field cultivation of *Lavandula angustifolia* with good antimicrobial activity and a rich composition of bioactive substances.

Based on the conducted research, it seems that lavender grown in southern Poland may be a useful plant for those industries that are looking for natural raw materials with effective antimicrobial and antioxidant properties. However, in order to confirm our initial assumptions, further analyses would be necessary to confirm how the climatic and soil conditions of Poland affect the variability of the analyzed features. Another important aspect is obtaining a high-quality extract. In our research, we obtained ethanol extracts, which economically seem to be less expensive than those obtained by traditional steam distillation, which is characterized by higher energy consumption. However, in future experiments, it would be necessary to assess the quality of the obtained raw material by isolating bioactive active substances from it and using modern extraction methods. In order for lavender grown in Poland to become a useful export raw material with good antimicrobial and antioxidant properties, further research is needed, with particular emphasis on the optimization of cultivation conditions and activity-controlled extraction.

## Figures and Tables

**Table 1:** Growth diameter of T. viride on a medium containing various amounts of lavender extracts.

Plant Material	Concentration of Extracts in Growth Medium (mL/100 mL)	Day of Observation			*p*-Value	α
		2	3	4		
		Diameter of Mycelium (mm)			Tukey’s Test	
ZO1	statistics F				1.02 × 10^−14^	0.05
	0 (control)	58.8	90.0	-	a	
	0.5	35.0	42.0	-	ab	
	1.0	5.7	5.7	-	b	
	2.5	5.0	5.0	-	b	
	5.0	5.0	5.0	-	b	
ZO2	statistics F				1.67 × 10^−8^	0.05
	0 (control)	58.8	90.0	-	a	
	0.5	21.5	23.0	-	b	
	1.0	6.0	6.0	-	b	
	2.5	5.0	5.0	-	b	
	5.0	5.0	5.0	-	b	

“a,b” is homogeneous groups by the Tukey test, *p*-value—probability of error, α—statistical significance level.

**Table 2:** Growth diameter of Ch. globosum on a medium containing various amounts of lavender extracts.

Plant Material	Concentration of Extracts in Growth Medium (mL/100 mL)	Day of Observation					*p*-Value	α
		2	3	5	7	9		
		Diameter of Mycelium (mm)					Tukey’s Test	
ZO1	statistics F						3.81 × 10^−8^	0.05
	0 (control)	16.0	20.3	28.8	40.2	44.8	a	
	0.5	6.0	6.0	6.0	6.0	6.0	b	
	1.0	5.0	5.0	5.0	5.0	5.0	b	
	2.5	5.0	5.0	5.0	5.0	5.0	b	
	5.0	5.0	5.0	5.0	5.0	5.0	b	
ZO2	statistics F						9.10 × 10^−8^	0.05
	0 (control)	16.0	20.3	28.8	40.2	44.8	a	
	0.5	5.2	6.3	6.3	6.7	6.7	b	
	1.0	5.0	5.0	5.0	5.0	5.0	b	
	2.5	5.0	5.0	5.0	5.0	5.0	b	
	5.0	5.0	5.0	5.0	5.0	5.0	b	

“a,b” is homogeneous groups by the Tukey test, *p*-value—probability of error, α—statistical significance level.

**Table 3:** Total phenolic content and antioxidant activity of lavender flowers (ZO1) and leaves (ZO2).

No.	Sample	Total Phenolic Content TPC	ABTS^•+^ RADICAL Scavenging Activity	DPPH^•^ Radical Scavenging Activity	Ferric Reducing Antioxidant Power Assay FRAP
		mg GAE/g	μmol Trolox Equivalent (TE)/g		
1	ZO1	19.78 ± 0.70 ^a^	168.86 ± 2.86 ^a^	97.47 ± 1.37 ^a^	62.88 ± 0.83 ^a^
2	ZO2	31.64 ± 0.85 ^b^	274.84 ± 0.74 ^b^	198.21 ± 1.34 ^b^	110.25 ± 1.25 ^b^

^a,b^ is homogeneous groups by the Tukey test.

**Table 4:** Analysis of variance in terms of the significance of the impact of ZO1 and ZO2.

	*p*-Value	X	Error
Total phenolic content	4.93 × 10^−5^	98.9	1.1
ABTS^•+^	4.03 × 10^−7^	99.9	0.1
DPPH^•^	8.70 × 10^−8^	99.9	0.1
FRAP	6.65 × 10^−7^	99.9	0.1

*p*—probability of error and X—percentage influence of factors (ZO1 or ZO2).

**Table 5:** Chemical composition of the headspace fraction (HS) of lavender flower extracts (ZO1).

No.	RT [min]	Peak Share in the Chromatogram [%]	Ordinary Substance Name	Systematic Substance Name	No. CAS
1	7.72	0.94	α-Pinene	2,6,6-Trimethylbicyclo [3.1.1]hept-2-ene	80-56-8
2	8.72	2.39	β-Pinene	6,6-Dimethyl-2-methylenebicyclo [3.1.1]heptane	127-91-3
3	9.04	4.79	Myrcene	7-Methyl-3-methylene-1,6-octadiene	123-35-3
4	9.45	1.97	3-Carene	3,7,7-Trimethylbicyclo [4.1.0]hept-3-ene	13466-78-9
5	9.73	1.61	m-Cymene (cymene isomers mix)	1-Isopropyl-3-methylbenzene	
6	9.83	7.09	Limonene	4-Isopropenyl-1-methyl-1-cyclohexene	5989-27-5
7	10.00	25.81	Ocimene isomers mix		
8	10.21	8.59	Ocimene isomers mix		
9	11.16	2.23	Linalool	3,7-Dimethyl-1,6-octadien-3-ol	78-70-6
10	11.36	2.02		1-Octen-3-yl acetate	2442-10-6
11	11.67	0.91		2,6-Dimethyl-2,4,6-octatriene	673-84-7
12	13.66	25.83	Linalyl acetate	3,7-Dimethyl-1,6-octadien-3-yl acetate	115-95-7
13	15.43	0.93	Lavandulyl acetate	5-Methyl-2-(1-methylethenyl)-4-hexylene-1-ol acetate	25905-14-0
14	16.03	3.99	α-santalene	6,7-dimethyl-7-(4-methylpent-3-enyl)-2,3,4,5-tetrahydro-1H-tricyclo [2.2.1.02,6]heptane	512-61-8
15	16.09	10.84	β-Caryophyllene	8-Methylene-4,11,11-trimethylbicyclo [7.2.0]undec-4-ene	87-44-5

**Table 6:** Calculated linear retention rates (ZO1).

Substance Name	α-Pinene	β-Pinene	Myrcene	3-Carene	m-Cymene	Limonene	Ocimene (Mix)	Ocimene (Mix)	Linalool	1-Octen-3-yl Acetate	2,6-Dimethyl-2,4,6-octatriene	Linalyl Acetate	Lavandulyl Acetate	α-Santalene	β-Caryophyllene
Molecular weight [g/mol]	136	136	196	136	134	136	136	136	154	170	136	196	196	204	204
Calculated Kovats retention index	933	964	991	1016	1023	1034	1050	1060	1100	1120	1130	1261	1288	1420	1425
Reference Kovats retention index *	926–1045	934–1138	986–1187	1004–1017	1010–1267	1031–1056	1043–1270	1043–1270	1082–1570	1097–1373	1129	1257–1569	1288–1597	1405–1574	1418–1657

* Reference Kovats retention index from: https://www.pherobase.com/ (access date: 20 August 2023).

**Table 7:** Chemical composition of the headspace fraction (HS) of lavender leaves extracts (ZO2).

No.	RT [min]	Peak Share in the Chromatogram [%]	Ordinary Substance Name	Systematic Substance Name	No. CAS
1	9.45	4.08	3-Carene	3,7,7-Trimethylbicyclo [4.1.0]hept-3-ene	13466-78-9
2	9.69	trace	p-Cymene (cymene isomers mix)	1-Isopropyl-4-methylbenzene	
3	9.74	trace	m-Cymene (cymene isomers mix)	1-Isopropyl-3-methylbenzene	
4	9.83	trace	Limonene	4-Isopropenyl-1-methyl-1-cyclohexene	5989-27-5
5	9.89	trace	Eucalyptol	1,3,3-Trimethyl-2-oxabicyclo [2.2.2]octane	470-82-6
6	14.66	4.70	silane		
7	16.03	12.12	α-santalene	6,7-dimethyl-7-(4-methylpent-3-enyl)-2,3,4,5-tetrahydro-1H-tricyclo [2.2.1.02,6]heptane	512-61-8
8	16.09	10.00	β-Caryophyllene	8-Methylene-4,11,11-trimethylbicyclo [7.2.0]undec-4-ene	87-44-5
9	16.27	23.19	Coumarin	1-Benzopyran-2-one	91-64-5
10	17.25	20.45	γ-cadinene	7-methyl-4-methylidene-1-propan-2-yl-2,3,4a,5,6,8a-hexahydro-1*H*-naphthalene	39029-41-9
11	19.67	19.85	7-Methoxycoumarin	Methyl umbelliferyl ether	531-59-9

**Table 8:** Calculated linear retention rates (ZO2).

Substance Name	3-Carene	p-Cymene	m-Cymene	Limonene	Eucalyptol	α-Santalene	β-Caryophyllene	Coumarin	γ-Cadinene	7-Methoxycoumarin
Molecular weight [g/mol]	136	134	134	136	154	204	204	146	204	176
Calculated Kovats retention index	1016	1020	1023	1034	1050	1420	1425	1429	1513	1660
Reference Kovats retention index *	1004–1017	1016–1303	1010–1267	1031–1056	1025–1224	1405–1574	1418–1657	1428–2465	1512–1819	1660–2981

* Reference Kovats retention index from: https://www.pherobase.com/ (access date: 20 August 2023).

**Table 9:** Chemical composition of phenol compounds.

No.	Compound	RT [min]	ZO1	ZO2	Type
			[µg/g]		
1	Syringic acid glucoside	1.933	70.15	328.10	phenolic acid
2	Caftaric acid	2.303	101.03	1110.18	phenolic acid
3	Ferulic acid glucosie I	3.081	<LOQ	<LOQ	phenolic acid
4	Coumaric acid glucoside I	3.121	671.26	797.42	phenolic acid
5	Caffeic acid	3.255	209.88	699.87	phenolic acid
6	Ferulic acid glucosie II	3.596	637.33	1156.45	phenolic acid
7	Isorhamnetin 3-*O*-rutinoside	3.66	<LOQ	<LOQ	flavonoid
8	Apigenin 4′-*O*-glucoside-7-*O*-glucuronide	3.829	146.58	170.02	flavonoid
9	Coumaric acid glucoside II	4.083	363.19	405.22	phenolic acid
10	Chicoric acid	4.397	221.68	79.83	phenolic acid
11	Ferulic acid glucosie III	4.563	813.23	1564.10	phenolic acid
12	Isorhamnetin 3-*O*-rhamnoside	4.775	123.10	1151.83	flavonoid
13	(+)Catechin-rhamnoside-pentoside	4.888	174.57	318.87	flavonoid
14	Salvinic acid B	5.305	87.09	97.26	phenolic acid
15	Apigenin C-glucoside	5.436	177.15	267.97	flavonoid
16	Rosmarinic acid	5.623	917.15	2547.31	phenolic acid
17	Ferulic acid	5.95	37.19	330.57	phenolic acid
18	Unidentified caffeic acid derivative	6.064	100.30	2826.27	phenolic acid
19	Kaempferol	6.696	92.23	821.82	flavonoid
20	Undefined caffeic acid derivative	7.036	102.62	4085.14	phenolic acid
21	Undefined caffeic acid derivative	7.333	55.05	131.71	phenolic acid
22	Apigenin	7.601	94.71	54.53	flavonoid

## Data Availability

No new data were created in these studies.

## Sample Availability

Samples of the extracts are available from the authors.
